# Visfatin and VEGF levels are not increased in adolescent girls with polycystic ovary syndrome

**DOI:** 10.3389/fendo.2024.1488249

**Published:** 2024-11-12

**Authors:** Karolina Skrzyńska, Agnieszka Zachurzok, Agnieszka Pietrusik, Karolina Jakubowska-Kowal, Aneta Gawlik-Starzyk

**Affiliations:** ^1^ Department of Pediatrics and Pediatric Endocrinology, School of Medicine in Katowice, Medical University of Silesia, Katowice, Poland; ^2^ Department of Pediatrics, Faculty of Medical Sciences in Zabrze, Medical University of Silesia, Zabrze, Poland

**Keywords:** polycystic ovary syndrome, cardiovascular diseases, visfatin, VEGF, adolescents

## Abstract

**Introduction:**

PCOS is one of the most commonly occurring endocrinopathies among women and increasingly affects adolescent populations. The connection between PCOS and various endocrinological, psychological, and CVD is increasingly recognized. Some studies have shown elevated levels of visfatin and VEGF among patients with PCOS, which are markers of vascular endothelial dysfunction. In our study, we evaluated the concentration of these parameters, focusing solely on a group of adolescents with PCOS, to assess whether these early markers of CVD are present at an early stage of diagnosis.

**Material and methods:**

In total, 80 adolescent girls participated in the study. 47 adolescents diagnosed with PCOS were included in the study group (mean age 15.68 ± 1.18 years, BMI 26.66 ± 6.41 kg/m2), while the remaining 33 regularly menstruating individuals (mean age 15.79 ± 1.22 years, BMI 25.44 ± 7.24 kg/m2) were assigned to the control group. Each participant underwent imaging, biochemical, and hormonal tests. Additionally, markers of endothelial dysfunction: VEGF and visfatin, were measured in all adolescents.

**Results:**

Both VEGF and visfatin levels did not differ significantly between PCOS and control group (p=0.30 and p=0.15, respectively). In the group of adolescent girls with PCOS, visfatin was significantly correlated with HDL, FSH, cortisol, and testosterone levels >55 ng/dl. VEGF was significantly correlated with fasting glucose, glucose levels after OGTT, estradiol, and waist circumference >80 cm.

**Conclusion:**

It can be indirectly inferred that both visfatin and VEGF should not be used as early markers for cardiometabolic complications among adolescent patients with PCOS. On the other hand, low visfatin levels, through their negative correlation with HDL, may have a protective effect on cardiovascular complications, while low VEGF levels, through their positive correlation with glucose levels, may have a protective influence on carbohydrate metabolism disorders.

## Introduction

Polycystic ovary syndrome (PCOS) is one of the most common endocrinopathies affecting women of reproductive age. The prevalence varies widely, ranging from 2.2% to 20%, depending on the diagnostic criteria and the studied population (1, 2). This condition increasingly affects adolescent populations, potentially affecting around 3-11% of adolescent girls (3–5). Rotterdam criteria (6) are commonly used for diagnosing PCOS, but they are applicable only to adult patients. Modified criteria proposed by Ibanez et al. (2017r) (7) and Teede et al. (2023) (8) have been developed for diagnosing PCOS in younger patients based on the presence of clinical or biochemical hyperandrogenism and menstrual irregularities.

PCOS is associated with a range of complications and comorbidities, including other endocrinological, psychological, and cardiometabolic disorders (9). Studies on adult populations have shown that PCOS is linked to an increased risk of hypertension (10), elevated triglyceride levels (11), decreased HDL cholesterol levels (12), and increased risks of non-fatal cerebrovascular disease events compared to women without PCOS (13). While researchers have extensively studied cardiometabolic disturbances in adult patients with PCOS, data regarding adolescents are still lacking. These diseases significantly impact the quality of life, highlighting the importance of multidisciplinary care, preventive education, and prompt diagnosis of comorbid conditions threatening the patient in adulthood.

Several studies have shown a significant link between classical cardiometabolic risk factors and PCOS (9–13). Additionally, increased levels of visfatin and vascular endothelial growth factor (VEGF), as early markers of endothelial dysfunction, have been noted in women with PCOS (14). Visfatin is suggested to induce the expression of proangiogenic factors, leading to endothelial dysfunction and increased cardiovascular risk (15). VEGF is a factor involved in angiogenesis and is secreted mainly in the situation of insufficient blood supply (16). VEGF is considered as a main modulator of angiogenesis in ovaries. Its expression is elevated in the stromal tissue of polycystic ovary (17). However, these studies were conducted on adult patients, and research on adolescents is still lacking. In our study, we aimed to examine whether increased levels of visfatin and VEGF, as early markers of cardiovascular disease, are present in adolescent girls with PCOS.

## Patients and methods

It was a prospective study involving a group of 80 adolescent girls aged 13 to 17 years. The study group comprised 47 patients who, upon comprehensive diagnostic evaluation, satisfied the complete set of criteria for a PCOS diagnosis as established by the 2017 Ibanez et al. consensus (7). Specifically, these individuals demonstrated:

1. Menstrual irregularities occurring more than 2 years after menarche, including oligomenorrhea (menstrual cycles less frequent than every 45 days), polymenorrhea (cycles shorter than 21 days), and secondary amenorrhea (absence of menstrual bleeding in the past 90 days);
2. Clinical hyperandrogenism (assessed based on excessive hair growth—significant hirsutism was defined as a Ferriman-Gallwey score of ≥8) or biochemical hyperandrogenism (diagnosed when total testosterone levels exceeded 55 ng/dL).

Exclusion criteria comprised eating disorders (anorexia nervosa, bulimia), hyperprolactinemia (prolactin [PRL] ≥721 mIU/l), adrenal disorders (17hydroxyprogesterone [17OHP] ≥10 ng/ml, in patients with 17OHP between 2.0-9.9 ng/ml, urine steroid profile results suggestive of congenital adrenal hyperplasia), and the use of medications known to influence sex steroids in the last 3 months (oral contraceptives, glucocorticosteroids, antiandrogens, aromatase inhibitors, metformin, anticonvulsants, etc.).

The control group consisted of 33 healthy, regularly menstruating girls, in whom PCOS was excluded.

Data regarding medical history, physical examination, and laboratory and imaging tests performed during hospitalization were collected based on medical documentation.

In all patients, the following data were analyzed:

- anthropometric measurements (body weight [kg], height [cm], BMI [kg/m2], age of menarche [years], hirsutism assessed according to the Ferriman-Gallwey scale [points], waist circumference [cm], hip circumference [cm], Waist-Hip Ratio (WHR), systolic pressure (mmHg), diastolic pressure (mmHg);
- biochemical tests results (alanine aminotransferase – ALT [U/l], aspartate aminotransferase – AST [U/l], total cholesterol - TC [mg/dl], triglycerides - TG [mg/dl], HDL cholesterol - HDL [mg/dl], LDL cholesterol - LDL [mg/dl], fasting glucose and at 120 minutes of oral glucose tolerance test (OGTT) after a load of 75 g glucose [mg/dl]), visfatin [ng/ml], vascular endothelial growth factor – VEGF [pg/ml]);
- hormonal tests results (total testosterone - T [ng/dl], estradiol - E [pmol/l], luteinizing hormone - LH [mIU/ml], follicle stimulating hormone - FSH [mIU/ml], dehydroepiandrosterone sulfate - DHEAS [µg/dl], androstenedione – A [ng/ml], fasting insulin and 120 minutes OGTT [µIU/ml]), cortisol [ug/dl]);
- pelvic ultrasound (5 MHz convex transducer, Siemens Medical Solution USA, Inc. apparatus) – ovarian volume [V];
- HOMA-IR (assessment of a model of homeostasis), and LH to FSH ratio (LH/FSH) were calculated.

Measurements of the concentrations of the tested hormones were made in the blood serum using the electrochemiluminescence method on the Cobas e411 apparatus (T, E), chemiluminescence on the Immulite 2000XPi apparatus (LH, FSH, DHEAS) or the ELISA method on the DS2 analyzer (17OHP, A) and ELISA (VEGF, visfatin).

Anthropometric data, biochemical, hormonal and immunological results were compared using the Statistica 14EN software. All values were expressed as mean standard deviation for normal or median (interquartile range) for skewed distribution. Comparison between groups was performed using t-Student test for normally distributed data and Mann-Whitney U test for non-normally distributed samples. Correlation analysis was performed using Pearson correlation coefficient for normally distributed, Spearman correlation coefficient for non-normally distributed data and Gamma correlation for categorized data.

P value <0.05 was considered statistically significant.

The study was performed according to the guidelines of the Helsinki Declaration on human experimentation and was approved by the Bioethics Committee of the Medical University of Silesia (PCN/0022/KB1/132/19). An informed consent was obtained from every subject or/and parent or guardian.

## Results

**Table 1** presents the clinical assessment of patients with PCOS and the control group. Clinically, there were no differences between the groups in terms of chronological age, age of menarche, and BMI. The hirsutism score tends to be higher in the PCOS group than in the control group.

**Table 1:** The clinical characteristics of PCOS patients and controls.

Variables	PCOS group (n=47)	Control group (n=33)	p-value
**Chronological age [years]**	15.68±1.18	15.79±1.22	0.64
**Age of menarche [years]**	12.06±1.68	12.48±1.55	0.53
**Waist circumference [cm]**	83.25±16.91	80.58±21.24	0.23
**Hip circumference [cm]**	99.13±9.23	97.94±12.01	0.28
**WHR**	0.83±0.09	0.81±0.11	0.21
**Hirsutism [points]**	5.00±5.79	2.67±5.28	0.06
**BMI [kg/m2]**	26.66±6.41	25.44±7.24	0.20
**Systolic blood pressure [mmHg]**	124.19±10.70	123.76±10.47	0.86
**Diastolic blood pressure [mmHg]**	74.57±7.95	74.28±8.63	0.94

BMI, body mass index; PCOS, polycystic ovary syndrome; WHR, waist-to-hip ratio.

Metabolic parameters were also analyzed, as illustrated in **Table 2**. A significantly lower concentration of HDL was found in the group of adolescent patients with PCOS compared to the control group. The remaining results did not differ between the groups.

**Table 2:** The biochemical and metabolic characteristics of PCOS patients and controls.

Variables	PCOS group (n=47)	Control group (n=33)	p-value
**ALAT [U/l]**	21.13±14.90	21.27±19.62	0.23
**ASPAT [U/l]**	23.19±11.19	23.58±13.00	0.71
**Glucose 0 [mg/dl]**	88.91±6.08	91.18±9.59	0.46
**Glucose 120 [mg/dl]**	112.66±29.80	116.81±20.92	0.23
**Insulin 0 [µIU/ml]**	15.00±5.55	15.10±7.15	0.78
**Insulin 120 [µIU/ml]**	87.60±38.20	68.70±25.65	0.17
**HOMA-IR**	3.22±1.29	3.24±1.51	0.72
**Total cholesterol [mg/dl]**	159.26±39.14	172.79±37.92	0.17
**HDL cholesterol [mg/dl]**	49.02±7.63	53.29±9.67	0.03
**LDL cholesterol [mg/dl]**	91.74±29.75	98.14±31.37	0.41
**Triglycerides [mg/dl]**	107.62±58.61	104.82±51.94	0.93

ALT, alanine aminotransferase; ASPAT, aspartate aminotransferase; Glucose 0, fasting glucose; Glucose 120, glucose 120 minutes OGTT; HOMA IR, assessment of a model of homeostasis; insulin 0, fasting insulin; insulin 120, insulin 120 minutes OGTT; PCOS, polycystic ovary syndrome.

The group of patients with PCOS was characterized by significantly higher concentrations of E, T, DHEAS, and A (p<0.01). The LH concentration was also higher in the study group, although with a trend towards statistical significance (p=0.08). Both VEGF and visfatin levels did not differ significantly between PCOS and the control group (p=0.30 and p=0.15, respectively). The hormonal assessment is presented in **Table 3**.

**Table 3:** The hormonal characteristics of PCOS patients and controls.

Variables	PCOS group (n=47)	Control group (n=33)	p-value
**LH [mIU/ml]**	8.90±5.51	7.14±2.61	0.09
**FSH [mIU/ml]**	5.20±1.95	5.21±1.92	0.10
**LH/FSH**	2.07±1.19	1.78±1.62	0.08
**Estradiol [pmol/l]**	175.60±104.65	139.50±61.35	0.04
**Testosteron [ng/dl]**	61.81±16.24	36.20±14.86	<0.01
**DHEAS [µg/dl]**	328.40±127.40	245.03±98.12	<0.01
**Androstendione [ng/ml]**	4.70±1.65	2.46±1.56	<0.01
**Visfatin [ng/ml]**	0.52±0.22	0.57±0.19	0.15
**VEGF [pg/ml]**	191.52±91.99	162.56±75.07	0.30

DHEAS, dehydroepiandrosterone sulfate; FSH, follicle stimulating hormone; LH, luteinizing hormone; MMP-9, matrix metalloproteinase-9; PCOS, polycystic ovary syndrome; VEGF, vascular endothelial growth factor.

In the group of patients with PCOS, visfatin levels showed a significant correlation with HDL, FSH, cortisol, and T >55 ng/dl (R=-0.35, p=0.01; R=-0.29, p=0.04; R=-0.31, p=0.03; R_ƴ_=-0.32, p=0.03, respectively). In the same group, significant correlations were found between VEGF levels and fasting glucose, glucose in OGTT, E, and waist circumference >80 cm (R=0.29, p=0.04; R=0.33, p=0.03; R=-0.38, p=0.01; R_ƴ_=0.30, p=0.03, respectively).

In the controls, visfatin levels were significantly correlated with T, DHEAS, LH/FSH ratio, elevated LDL, and T >55 ng/dl (R=-0.42, p=0.01; R=-0.38, p=0.03; R=-0.36, p=0.04; R_ƴ_=0.55, p=0.03; R_ƴ_=-0.53, p=0.04, respectively). In the same group, the only significant correlation was found between VEGF levels and DHEAS (R=-0.35, p=0.04).

## Discussion

PCOS is a syndrome that increases cardiometabolic risk in women regardless of its phenotype. Among these patients, there is a higher incidence of conditions such as hypertension, hypertriglyceridemia, and metabolic syndrome (18, 19). Increased levels of visfatin and vascular endothelial growth factor (VEGF), as early markers of endothelial dysfunction, have been noted in women with PCOS (20). Because research on adolescents with PCOS is still lacking, we aimed to examine whether increased levels of visfatin and VEGF, as early markers of cardiovascular disease, are present in adolescent girls with PCOS. We found no significant difference in terms of visfatin and VEGF levels between the PCOS and control group. However, visfatin level correlated negatively in PCOS girls with HDL level and hyperandrogenemia. While VEGF was related to glucose levels and central obesity.

Studies in the adult female population with PCOS have shown that visfatin is positively correlated with cardiometabolic risk factors (14). Due to its sensitivity and specificity, visfatin has been suggested as potentially useful in predicting this risk among PCOS patients. Other studies have demonstrated increased visfatin levels in patients with PCOS, which are associated with the pathophysiology of this syndrome and the potential for metabolic complications (21).

Analyzing visfatin levels in our study among adolescents with PCOS, we found that visfatin levels were similar in both groups. Cekmez et al. (22) obtained results similar to ours. In contrast, Wang Y et al. (23) and Dambala et al. (20) reported higher visfatin levels in the PCOS patient group compared to controls. The differences in results can be attributed to the varying characteristics of the study groups. The study by Cekmez et al. (22) involved adolescent PCOS patients whose clinical characteristics were similar to our patients, whereas the other studies focused on adult women. In our study of adolescents with PCOS, visfatin was negatively correlated with HDL levels, with no association with other lipid parameters. A similar correlation was found in the study by Kim J. et al. (24), conducted on a group of lean, adult women with PCOS. The mechanism explaining this correlation may be the pro-inflammatory properties of visfatin, which affects the regulation of interleukins such as Interleukin 6 (IL-6), Interleukin 1B (IL-1B), and tumor necrosis factor alpha (TNF-α) (25). It has been demonstrated that TNF-α reduces the levels of apolipoprotein A-I (apo A-I) and apolipoprotein B (apo B). Apo A-I is a key protein in HDL, thus its deficiency contributes to a decrease in the total HDL cholesterol pool (26). Additionally, through another mechanism, TNF-α contributes to the reduction of HDL levels by increasing the activity of hepatic lipase (HL), an enzyme that hydrolyzes triglycerides and phospholipids contained in HDL (27). However, conclusions from other studies differ and indicate a positive correlation between visfatin levels and HDL among women with PCOS (Ruan et al. (28), Wang et al. (23)). The limited number of studies on adolescents with PCOS does not allow for precise analysis and reliable comparison of the research. Adolescent patients with PCOS are at the early stages of the disease, which undoubtedly affects their distinct metabolic profile.

Regarding VEGF, studies in the adult population have shown that susceptibility to PCOS may depend on VEGF polymorphisms and ethnicity, specifically among Asian or Caucasian populations. Certain polymorphisms, such as VEGF rs2010963, decreases the risk of PCOS in the general population, whereas VEGF rs3025039 may decrease the risk of PCOS in Asian populations but increase it in Caucasian populations (29). Other studies found no association between the risk of PCOS and certain VEGF polymorphisms in the general population (30). Research on VEGF levels in adult PCOS patients showed significantly higher levels in the PCOS group compared to controls, with VEGF levels dependent on BMI (31). In our study, VEGF levels were lower in the adolescent PCOS group compared to the control group, but this difference was not statistically significant. Our findings were not confirmed by other researchers, who demonstrated higher VEGF concentrations in the population of adult women with PCOS compared to the control group [Dambala et al. (20), Simkova et al. (32), Elci E (33), Tahergorabi et al. (31), Abd El Aal DE et al. (34)]. The differing results may be attributed to the young age of the patients who participated in our study. It is well-established that factors such as metabolic activity, inflammation, tissue damage, angiogenesis, lifestyle, etc., influence VEGF levels. Differences in the interaction of VEGF with specific receptors may also result from hormonal changes and insulin resistance, which are age-dependent. Additionally, genetic factors also play a role in regulating VEGF levels, as demonstrated by studies conducted among women from Bahrain (35).

VEGF, through interaction with specific receptors, activates the phosphatidylinositol 3-kinase (PI3K) and protein kinase AKT signaling pathways, as well as the mitogen-activated protein kinase (MAPK) pathway (36). This leads to alterations in insulin signaling pathways. As a result of these processes, glucose is not effectively utilized by muscle or adipose tissue due to reduced uptake by these tissues, consequently contributing to hyperglycemia. Furthermore, VEGF can influence the expression and function of glucose transporters, particularly GLUT4. Dysregulation of GLUT4 function results in impaired glucose uptake by muscle and adipose cells, contributing to hyperglycemia through an additional mechanism (37). In our study VEGF was significantly correlated with fasting glucose and glucose levels in OGTT, indirectly suggesting a role for VEGF in reducing the risk of carbohydrate metabolism disorders among adolescent PCOS patients. The lack of other studies on VEGF levels in adolescent patients prevents the formulation of reliable conclusions.

Limitation of our study is the insufficient sample size to stratify the cohort into distinct experimental and control groups based on BMI status. A longitudinal study would be particularly valuable to determine if the observed associations and differences persist into adulthood, especially considering the duration of the disease.

## Conclusions

Low levels of visfatin and VEGF among adolescents with PCOS may be due to the early stage of the disease, and at this stage, they should not be used as early markers of metabolic risk. On the other hand, the observed negative correlations between visfatin levels and HDL, as well as positive correlations between VEGF levels and glucose among adolescents with PCOS, could indirectly serve as protective markers against cardiovascular diseases and diabetes.

It is crucial to conduct further studies on girls with PCOS to better understand their cardiometabolic risk and to explore markers that can help identify patients at the highest risk of complications. This would enable timely interventions such as lifestyle changes, appropriate diets, and proper treatment. Given the prevalence of PCOS and its consequences for metabolic, reproductive, and mental health, the occurrence of this syndrome in adolescent patients should be carefully studied. The continuation of research in this patient population and the conducting of studies on larger groups are necessary for a more precise understanding of the problem.

## Data Availability

The raw data supporting the conclusions of this article will be made available by the authors, without undue reservation.

## References

[1] Boyle JA Cunningham J O'Dea K Dunbar T Norman RJ. Prevalence of polycystic ovary syndrome in a sample of Indigenous women in Darwin, Australia. Med J Aust. (2012) 196:62–6. doi: 10.5694/mja11.10553 22256938

[2] Ma YM Li R Qiao J Zhang XW Wang SY Zhang QF. Characteristics of abnormal menstrual cycle and polycystic ovary syndrome in community and hospital populations. Chin Med J (Engl). (2010) 123:2185–9. doi: 10.3760/cma.j.issn.0366-6999.2010.16.005 20819662

[3] Hickey M Doherty DA Atkinson H Sloboda DM Franks S Norman RJ. Clinical, ultrasound and biochemical features of polycystic ovary syndrome in adolescents: implications for diagnosis. Hum Reprod. (2011) 26:1469–77. doi: 10.1093/humrep/der102 21478180

[4] Hashemipour M Faghihimani S Zolfaghary B Hovsepian S Ahmadi F Haghighi S. Prevalence of polycystic ovary syndrome in girls aged 14-18 years in Isfahan, Iran. Horm Res. (2004) 62:278–82. doi: 10.1159/000081842 15523185

[5] Asgharnia M Mirblook F Soltani M. The prevalence of polycystic ovary syndrome (PCOS) in high school students in Rasht in 2009 according to NIH criteria. Int J Fertil Steril. (2011) 4:156–9. doi: 10.18502/ijrm.v17i8.4818 PMC4023501 24851175

[6] Rotterdam ESHRE/ASRM-Sponsored PCOS Consensus Workshop Group. Revised 2003 consensus on diagnostic criteria and long-term health risks related to polycystic ovary syndrome (PCOS). Hum Reprod. (2004) 19:41–7. doi: 10.1093/humrep/deh098 14688154

[7] Ibáñez L Oberfield SE Witchel S Auchus RJ Chang RJ Codner E. An international consortium update: pathophysiology, diagnosis, and treatment of polycystic ovarian syndrome in adolescence. Horm Res Paediatr. (2017) 88:371–95. doi: 10.1159/000479371 29156452

[8] Teede HJ Tay CT Laven JJE Dokras A Moran LJ Piltonen TT. Recommendations from the 2023 international evidence-based guideline for the assessment and management of polycystic ovary syndrome. J Clin Endocrinol Metab. (2023) 108:2447–69. doi: 10.1210/clinem/dgad463 PMC10505534 37580314

[9] Wekker V van Dammen L Koning A Heida KY Painter RC Limpens J. Long-term cardiometabolic disease risk in women with PCOS: a systematic review and meta-analysis. Hum Reprod Update. (2020) 26:942–60. doi: 10.1093/humupd/dmaa029 PMC7600286 32995872

[10] Cibula D Cífková R Fanta M Poledne R Zivny J Skibová J. Increased risk of non-insulin dependent diabetes mellitus, arterial hypertension and coronary artery disease in perimenopausal women with a history of the polycystic ovary syndrome. Hum Reprod. (2000) 15:785–9. doi: 10.1093/humrep/15.4.785 10739820

[11] Carmina E Campagna AM Lobo RA. Emergence of ovulatory cycles with aging in women with polycystic ovary syndrome (PCOS) alters the trajectory of cardiovascular and metabolic risk factors. Hum Reprod. (2013) 28:2245–52. doi: 10.1093/humrep/det119 23595974

[12] Talbott EO Zborowski JV Rager JR Kip KE Xu X Orchard TJ. Polycystic ovarian syndrome (PCOS): a significant contributor to the overall burden of type 2 diabetes in women. J Womens Health (Larchmt). (2007) 16:191–7. doi: 10.1089/jwh.2006.0098 17388735

[13] Merz CN Shaw LJ Azziz R Stanczyk FZ Sopko G Braunstein GD. Cardiovascular disease and 10-year mortality in postmenopausal women with clinical features of polycystic ovary syndrome. J Womens Health (Larchmt). (2016) 25:875–81. doi: 10.1089/jwh.2015.5441 PMC5311460 27267867

[14] Dambala K Paschou SA Michopoulos A Siasos G Goulis DG Vavilis D. Biomarkers of endothelial dysfunction in women with polycystic ovary syndrome. Angiology. (2019) 70:797–801. doi: 10.1177/0003319719840091 30969784

[15] Garten A Schuster S Penke M Gorski T de Giorgis T Kiess W. Physiological and pathophysiological roles of NAMPT and NAD metabolism. Nat Rev Endocrinol. (2015) 11:535–46. doi: 10.1038/nrendo.2015.117 26215259

[16] Melincovici CS Boşca AB Şuşman S Mărginean M Mihu C Istrate M. Vascular endothelial growth factor (VEGF) - key factor in normal and pathological angiogenesis. Rom J Morphol Embryol. (2018) 59:455–67. 30173249

[17] Heinolainen K Karaman S D'Amico G Tammela T Sormunen R Eklund L. VEGFR3 modulates vascular permeability by controlling VEGF/VEGFR2 signaling. Circ Res. (2017) 120:1414–25. doi: 10.1161/CIRCRESAHA.116.310477 PMC6959003 28298294

[18] Teede H Deeks A Moran L. Polycystic ovary syndrome: a complex condition with psychological, reproductive and metabolic manifestations that impacts on health across the lifespan. BMC Med. (2010) 8:41. doi: 10.1186/1741-7015-8-41 20591140 PMC2909929

[19] Profili NI Castelli R Gidaro A Manetti R Maioli M Petrillo M. Possible effect of polycystic ovary syndrome (PCOS) on cardiovascular disease (CVD): an update. J Clin Med. (2024) 13:698. doi: 10.3390/jcm13030698 38337390 PMC10856325

[20] Dambala K Vavilis D Bili E Goulis DG Tarlatzis BC. Serum visfatin, vascular endothelial growth factor and matrix metalloproteinase-9 in women with polycystic ovary syndrome. Gynecol Endocrinol. (2017) 33:529–33. doi: 10.1080/09513590.2017.1296425 28300464

[21] Al- Ghazali BS Mohammed AA Fahad AM. The association of serum visfatin in women with polycystic ovary syndrome: A case-control study. Revis Bionatura. (2022) 7:60. doi: 10.21931/RB/2022.07.04.60

[22] Cekmez F Cekmez Y Pirgon O Canpolat FE Aydinöz S Metin Ipcioglu O. Evaluation of new adipocytokines and insulin resistance in adolescents with polycystic ovary syndrome. Eur Cytokine Netw. (2011) 22:32–7. doi: 10.1684/ecn.2011.0279 21411410

[23] Wang Y Yu P. Clinical significance and changes of serum visfatin, adiponectin and leptin levels in patients with polycystic ovarian syndrome. Zhong Nan Da Xue Xue Bao Yi Xue Ban. (2009) 34:72–5. 19197132

[24] Kim JJ Choi YM Hong MA Kim MJ Chae SJ Kim SM. Serum visfatin levels in non-obese women with polycystic ovary syndrome and matched controls. Obstet Gynecol Sci. (2018) 61:253–60. doi: 10.5468/ogs.2018.61.2.253 PMC5854906 29564317

[25] Wu MH Tsai CH Huang YL Fong YC Tang CH. Visfatin Promotes IL-6 and TNF-α Production in Human Synovial Fibroblasts by Repressing miR-199a-5p through ERK, p38 and JNK Signaling Pathways. Int J Mol Sci. (2018) 19:190. doi: 10.3390/ijms19010190 29316707 PMC5796139

[26] Popa C Netea MG van Riel PL van der Meer JW Stalenhoef AF. The role of TNF-alpha in chronic inflammatory conditions, intermediary metabolism, and cardiovascular risk. J Lipid Res. (2007) 48:751–62. doi: 10.1194/jlr.R600021-JLR200 17202130

[27] Feingold KR Soued M Staprans I Gavin LA Donahue ME Huang BJ. Effect of tumor necrosis factor (TNF) on lipid metabolism in the diabetic rat. Evidence that inhibition of adipose tissue lipoprotein lipase activity is not required for TNF-induced hyperlipidemia. J Clin Invest. (1989) 83:1116–21. doi: 10.1172/JCI113991 PMC303797 2703526

[28] Ruan X Li M Min M Ju R Wang H Xu Z. Plasma visfatin and apelin levels in adolescents with polycystic ovary syndrome. Gynecol Endocrinol. (2023) 39:2216807. doi: 10.1080/09513590.2023.2216807 37248950

[29] Zhao J Li D Tang H Tang L. Association of vascular endothelial growth factor polymorphisms with polycystic ovarian syndrome risk: a meta-analysis. Reprod Biol Endocrinol. (2020) 18:18. doi: 10.1186/s12958-020-00577-0 32164758 PMC7069028

[30] Li Y Fang L Yu Y Shi H Wang S Li Y. Association between vascular endothelial growth factor gene polymorphisms and PCOS risk: a meta-analysis. Reprod BioMed Online. (2020) 40:287–95. doi: 10.1016/j.rbmo.2019.10.018 31956063

[31] Tahergorabi Z Salmani F Jonaidabad SH Behdani B Yazdi P Zardast M. Association of serum levels of vascular endothelial growth factor and thrombospondin-1 to body mass index in polycystic ovary syndrome: a case-control study. Obstet Gynecol Sci. (2019) 62:420–8. doi: 10.5468/ogs.2019.62.6.420 PMC6856488 31777738

[32] Šimková M Vítků J Kolátorová L Vrbíková J Vosátková M Včelák J. Endocrine disruptors, obesity, and cytokines - how relevant are they to PCOS? Physiol Res. (2020) 69:S279–93. doi: 10.33549/physiolres.934521 PMC8603732 33094626

[33] Elci E Kaya C Cim N Yildizhan R Elci GG. Evaluation of cardiac risk marker levels in obese and non-obese patients with polycystic ovaries. Gynecol Endocrinol. (2017) 33:43–7. doi: 10.1080/09513590.2016.1203893 27425379

[34] Abd El Aal DE Mohamed SA Amine AF Meki AR. Vascular endothelial growth factor and insulin-like growth factor-1 in polycystic ovary syndrome and their relation to ovarian blood flow. Eur J Obstet Gynecol Reprod Biol. (2005) 118:219–24. doi: 10.1016/j.ejogrb.2004.07.024 15653207

[35] Almawi WY Saldanha FL Mahmood NA Al-Zaman I Sater MS Mustafa FE. Relationship between VEGFA polymorphisms and serum VEGF protein levels and recurrent spontaneous miscarriage. Hum Reprod. (2013) 28:2628–35. doi: 10.1093/humrep/det308 23900206

[36] Ruan GX Kazlauskas A. VEGF-A engages at least three tyrosine kinases to activate PI3K/Akt. Cell Cycle. (2012) 11:2047–8. doi: 10.4161/cc.20535 PMC3368856 22647379

[37] Zhang X Lu JJ Abudukeyoumu A Hou DY Dong J Wu JN. Glucose transporters: Important regulators of endometrial cancer therapy sensitivity. Front Oncol. (2022) 12:933827. doi: 10.3389/fonc.2022.933827 35992779 PMC9389465

